# Selective Cytotoxicity of Amidinopiperidine Based Compounds Towards Burkitt’s Lymphoma Cells Involves Proteasome Inhibition

**DOI:** 10.1371/journal.pone.0041961

**Published:** 2012-07-30

**Authors:** Martina Gobec, Ales Obreza, Matevz Prijatelj, Boris Brus, Stanislav Gobec, Irena Mlinaric-Rascan

**Affiliations:** University of Ljubljana, Faculty of Pharmacy, Ljubljana, Slovenia; Stanford University, United States of America

## Abstract

Serine proteases have proven to be promising pharmacological targets in contemporary drug discovery for cancer treatment. Since azaphenylalanine-based compounds manifest cytotoxic activity, we have selected serine protease inhibitors designed and synthesized in-house with large hydrophobic naphthalene moiety for screening. The cytotoxic potential of screened molecules was correlated to modifications of R^1^ residues. The most cytotoxic were compounds with greater basicity; amidinopiperidines, piperidines and benzamidines. Amidinopiperidine-based compounds exert cytotoxicity in low µM range, with IC_50_ 18 µM and 22 µM for inhibitors **15** and **16** respectively. These compounds exhibited selective cytotoxicity towards the Burkitt’s lymphoma cells Ramos and Daudi, and proved nontoxic to PMBC, Jurkat and U937. They induce caspase-dependent apoptotic cell death, as demonstrated by the use of a pan-caspase inihibitor, zVADfmk, which was able to rescue Ramos cells from compound(s)-induced apoptosis. We confirm a disruption of the pro-survival pathway in Burkitt’s lymphoma through NFκB inhibition. The accumulation of phosphorylated precursor (p105) and inhibitory (IκB) molecules with no subsequent release of active NFκB implicated the involvement of proteasome. Indeed, we show that the amidinopiperidine-based compounds inhibit all three proteolytical activities of the human 20S proteasome, with the most prominent effect being on the trypsin-like activity. Consistently, treatment of Ramos cells with these compounds led to an increase in ubiquitinated proteins. The amidinopiperidine-based serine protease inhibitors presented are, as selective inducers of apoptosis in Burkitt’s lymphoma cells, promising leads for the development of novel chemotherapeutics.

## Introduction

Apoptosis is a natural process essential for multicellular development and the maintenance of tissue homeostasis. The deregulation of apoptosis disrupts the fine balance between cell proliferation and cell death, thus leading to diseases such as cancer. The development of drugs able to restore cell death may therefore be an effective approach in the treatment of cancer [1], [2]. Numerous proteins, including protein kinases, signalling adapters and proteases, have proven to be effective targets. Novel targets also comprise non-caspase proteases such as serine proteases, which have been reported to play an important role in the initiation or propagation of programmed cell death; however, the underlying molecular mechanisms have not yet been fully investigated and elucidated [3], [4], [5], [6], [7]. Serine proteases form a large family of proteolytic enzymes involved in numerous biological processes and can be divided into three subgroups, depending on the specific substrate cleavage; the chymotrypsin-, trypsin- and elastase-like serine proteases. Only a limited number of serine proteases have been identified as actively participating in the process of cell death, among them granzymes A and B, HtrA2/Omi, apoptotic protein 24 (AP24) and tissue-type plasminogen activator (tPA) [8], [9], [10]. Studies with synthetic and endogenous serine protease inhibitors (serpins) have revealed that serine proteases may act as both the pro- and anti-apoptotic molecules [11], [12].

Many cancer cells, especially hematopoietic malignancies, achieve resistance to radiotherapy or chemotherapy through mutations of key molecules in the nuclear factor kappa B (NFκB) signalling pathway that leads to its constitutive activation [13], [14]. Thus, targeting NFκB in malignancies that depend on this pro-survival signal is known to lead to apoptosis. The main step in NFκB activation is the phosphorylation of the precursor molecules (*e.g.* p100 and p105) and inhibitory proteins (*e.g.* IκB), which are subsequently ubiquitinated and proteolytically degraded by the proteasome. This, in turn, leads to the release of NFκB homo- and hetero-dimers (NFκB1, NFκB2, p50/p65), which translocate into the nucleus where they bind with the promoter of NFκB target genes [14], [15], [16]. *N*-tosyl-l-phenylalanine chloromethyl ketone (TPCK) is a specific chymotrypsin-like serine protease inhibitor that has been used in several studies investigating cell death. In some model systems it was shown to induce apoptosis, which effect was proposed to have been mediated through the reduction of NFκB activity [17], [18], [19], [20], [21].

One of the major pharmacological strategies used to target NFκB activity is the inhibition of the proteasome. The proteasome is a multi-subunit protein with at least three different activities, referred to as the chymotrypsin-, trypsin- and caspase-like peptidases. Proteasome inhibition is already successfully used in the treatment of multiple myelomas, where a dipeptide boronic acid derivative, bortezomib, is used [22], [23].

The above indicates that the inhibition of serine proteases may be a promising pharmacological approach in the treatment of malignant diseases. In the search for novel inducers of apoptosis we have assayed selected serine protease inhibitors possessing *N'*-acyl-2-naphthohydrazide or *N'*-acyl-naphthalene-2-sulfonohydrazide moiety, since we have previously shown that azaphenylalanine-based compounds show cytotoxic activity and concluded that a large hydrophobic naphthalene ring is crucial for the observed effect [24]. Herein we report on structurally related amidinopiperidine-based compounds exhibiting selective cytotoxic properties towards Burkitt’s lymphoma cells that trigger caspase-dependent apoptosis. A more detailed analysis of the molecular mechanism revealed that these compounds decrease NFκB activity through the inhibition of proteasome activity.

## Results

### Selection Criteria for Serine Protease Inhibitors Include Large Hydrophobic Moiety

**Figure 1:**
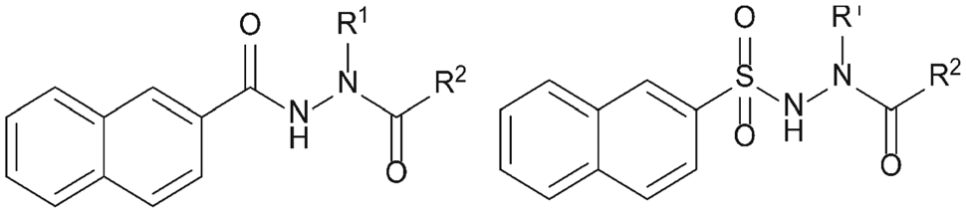
General structure of compounds. All compound possess *N'*-acyl-2-naphthohydrazide or *N'*-acyl-naphthalene-2-sulfonohydrazide moiety.

**Figure 2:**
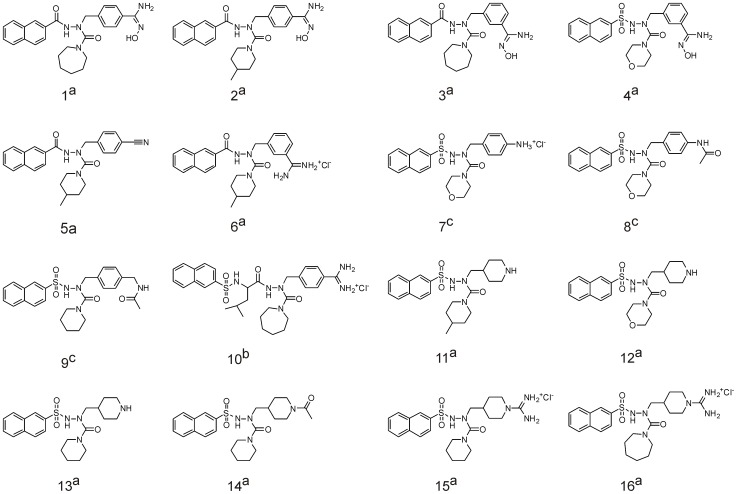
Structural formulae of biologically evaluated compounds. References with description of compound synthesis; **^a^** [27], **^b^** [26], **^c^** [25].

Our former investigations of apoptosis-inducing serine protease inhibitors revealed the cytotoxic effects of azaphenylalanine-based entities. In search for novel agents that are specifically cytotoxic for B-cell lymphomas, we have chosen to evaluate a new group of compounds. The general structure of the selected inhibitors (**1–16**) is presented in Figure 1 and consists of the relatively rigid *N'*-acyl-2-naphthohydrazide or *N'*-acyl-naphthalene-2-sulfonohydrazide moiety. The selected compounds contain a large hydrophobic naphthalene moiety since we have previously demonstrated its importance in inducing apoptosis [24]. Compounds **1–10** have a substituted benzyl group, while the rest of the compounds (**11–16**) are derivatives of piperidine. All 16 compounds possess inhibitory properties against trypsin, thrombin, factor Xa, and/or α-chymotrypsin. The ability of tested compounds **1–16** to inhibit α-chymotrypsin was determined using Suc-Ala-Ala-Pro-Phe-AMC as a substrate. The inhibition of the enzymatic activity of thrombin, trypsin and factor Xa had been determined previously with amidolytic enzyme assays using chromogenic substrates as described in the references listed in Figure 2 ([25], [26], [27]). The K_i_ values for the selected compounds are summarized in Table 1.

**Table 1:** K_i_ values of tested compounds for thrombin, factor Xa, trypsin and α-chymotrypsin.

Inhibitor	Ki (µM)			
	Thrombin	Factor Xa	Trypsin	α-chymotrypsin
**1**	5,2	78	660	33,5
**2**	>200	>200	200	259,3
**3**	4,8	60	>100	3
**4**	3,1	60	48	41
**5**	ND	ND	ND	273,1
**6**	6,8	5,7	1,8	17,1
**7**	4,5	62	5,6	4
**8**	ND	ND	ND	24,7
**9**	ND	ND	ND	40,6
**10**	10	>100	>100	8,8
**11**	19	ND	>200	38,8
**12**	53	ND	>200	149,9
**13**	30	ND	>200	65,9
**14**	ND	ND	ND	47,7
**15**	0,47	ND	39	27,6
**16**	0,5	ND	66	28,7

ND, not determined.

### Selective Cytotoxicity of Amidinopiperidine Compounds Towards Burkitt’s Lymphoma Cells

**Figure 3:**
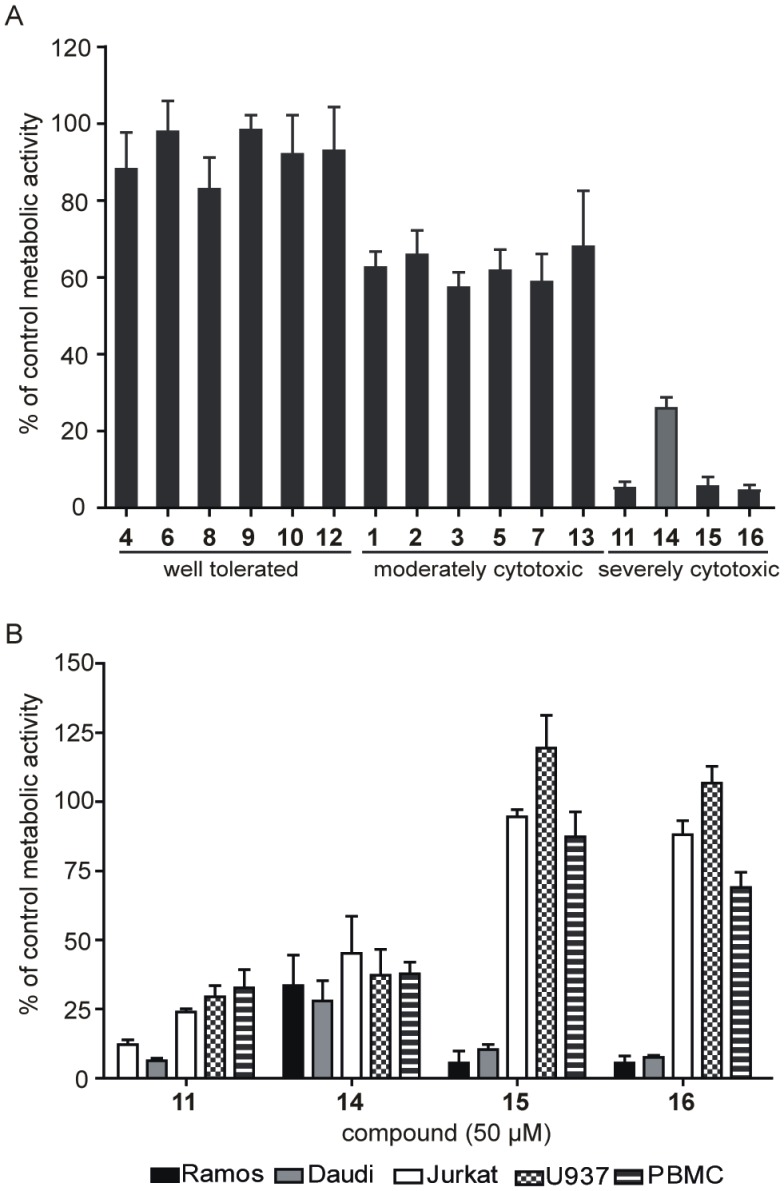
The cytotoxicity screening of serine protease inhibitors. (A) Ramos cells (1×10^5^ cells/ml) were incubated in the presence of inhibitors at 100 µM (**1**–**13**, **15**, **16**) or 50 µM (**14**) for 24 h. (B) PBMC (1×10^6^ cells/ml), Jurkat, Daudi, Ramos and U937 (10^5^ cells/ml) were incubated for 24 h with 50 µM of compounds. Data present the residual metabolic activity as a percentage relative to control cells incubated in growth media supplemented with DMSO vehicle (mean ± SD) from three independent experiments, each conducted in triplicate.

When investigating the potency of selected serine protease inhibitors to induce cell death in human B lymphoma cells, we identified a set of compounds cytotoxic for Burkitt’s lymphoma. Using a metabolic activity assay (MTS), we evaluated the proliferation rate of Ramos Burkitt’s lymphoma cells, in the presence of serine protease inhibitors. Cells were treated for 24 h with the compound of interest at concentrations of 100 µM (**1**–**13, 15, 16**) or 50 µM (**14**). After a comparison of metabolic activity relative to that in the untreated control, three groups with distinct modes of action were determined (Figure 3A). The first group (compounds **4**, **6**, **8**–**10** and **12)** were well tolerated by Ramos, since their residual metabolic activity did not fall below 80%. The second group (compounds **1**–**3**, **5**, **7** and **13)** caused a moderate decrease (50% to 80%) relative to untreated cells. The last group of compounds (**11**, **15**, **16**) severely impaired the metabolic activity, such that it fell to under 6%. Compound **14** was used at 50 µM due to poor solubility and led to a residual metabolic activity of 26%.

**Figure 4:**
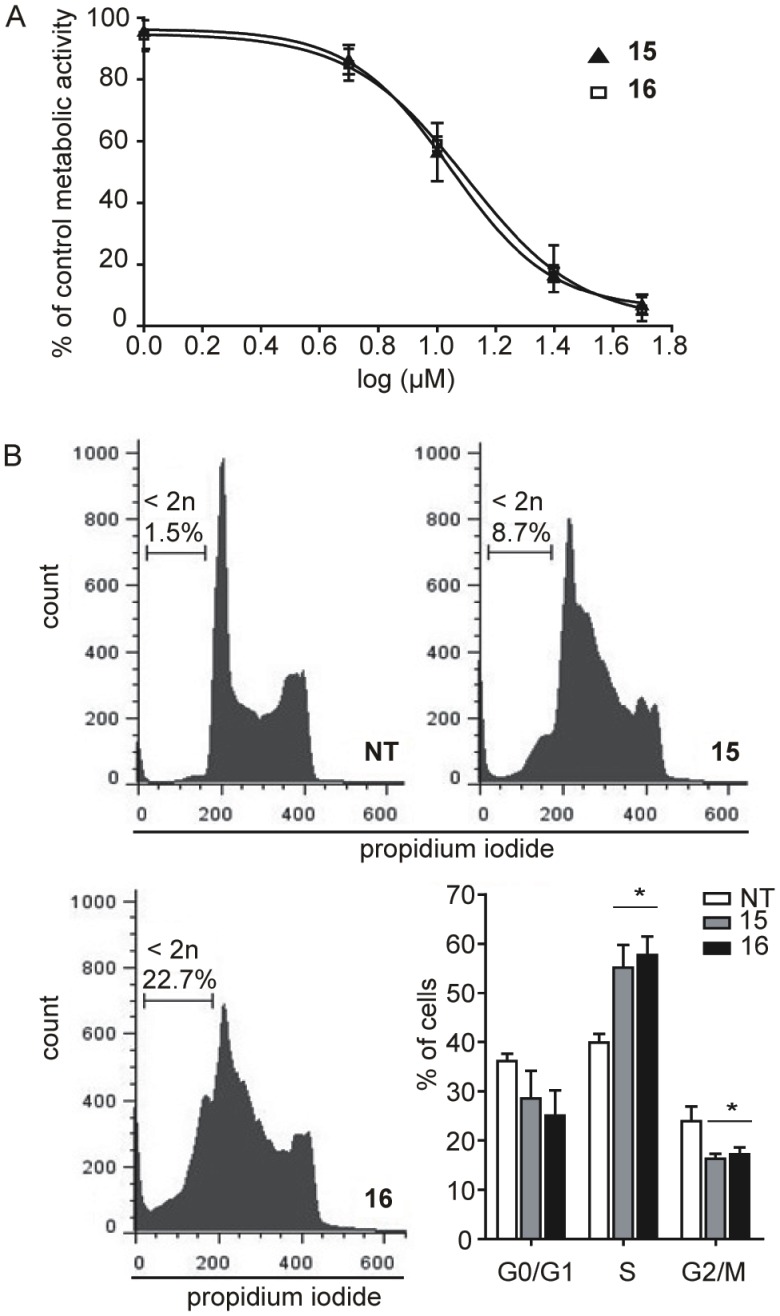
Amidinopiperidine-based compounds exert concentration-dependent cytotoxicity. (A) Metabolic activity assay. Ramos cells (1×10^5^ cells/ml) were incubated with different concentrations of compounds **15** and **16** (1 µM, 5 µM, 10 µM, 25 µM and 50 µM) for 24 h. (B) Cell cycle analysis. Ramos cells were incubated with 50 µM compound of interest for 24 h. PI staining was performed. The percentage of cells in the sub-G1 phase and cell cycle distribution of total viable cells are shown. * P<0.05; NT, non-treated cells.

Compounds **11**, and **14**–**16** were further tested for selective cytotoxicity. The experiments were conducted on freshly isolated primary peripheral blood mononuclear cells (PBMC) and three other haematological cancers: human T cell leukaemia (Jurkat), human histiocytic lymphoma (U937) and human Burkitt’s lymphoma (Daudi). An MTS assay was performed following 24 h of treatment with the compound of interest at 50 µM. We compared the percentages of residual metabolic activity between different cell types and determined that serine protease inhibitors **15** and **16** caused a severe and selective decrease in Burkitt’s lymphoma cells Ramos and Daudi only, with no or only moderate decreases being observed in the cases of PBMC, Jurkat and U937. Compounds **11** and **14** displayed a similar cytotoxic effect in all cell types, with residual metabolic activity under 40% (Figure 3B). The inhibition of proliferation in Ramos cells, mediated with compounds **15** and **16,** was concentration dependent. The IC_50_ values were determined using GraphPad Prism5, with results of 18 µM and 22 µM for inhibitors **15** and **16** respectively (Figure 4A).

Cell cycle analysis of Ramos cells treated for 24 h with 50 µM of **15** or **16** revealed an accumulation of cells in the S phase of the cycle and an increase of cell mortality relative to untreated cells (Figure 4B). The number of hypodiploid cells, measured at 3.4% in untreated cells, increased following 48 h treatment to 42.5% and 51.1% for compounds **15** and **16** respectively; thus, the decrease in metabolic activity was attributed to increased cell mortality.

### Apoptotic Cell Death is Caspase-dependent

**Figure 5:**
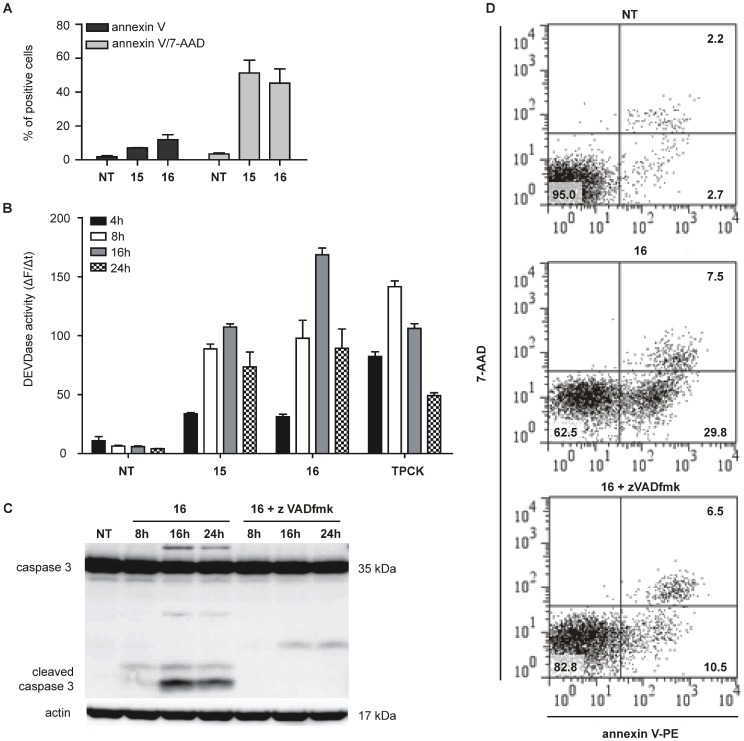
zVADfmk inhibits amidinopiperidine-based compounds induced apoptosis. (A) The determination of Annexin V and 7-AAD positive Ramos cells treated with compounds **15** or **16** for 24 h. The data present the percentage of gated cells. (B) Caspase 3/7 activity was determined in cell lysates of Ramos cells treated for 4 h, 8 h, 16 h and 24 h with either 50 µM inhibitor or 10 µM TPCK, used as a positive control. Cleavage of Ac-DEVD-AFC in whole cell lysates was determined spectrofluorometrically. The results are presented as changes in fluorescence as a function of time. (C) Western blot analysis of the caspase-3 processing. Cells were treated for indicated time periods in the presence of zVADfmk (50 µM) and/or compound 16 (50 µM). (D) Analysis of Annexin V/7-AAD positive cells after **16** h treatment with compound **16** in the absence or presence of zVADfmk. NT, non-treated cells.

To explore the mode of cell death induced by the two selectively cytotoxic serine protease inhibitors **15** and **16**, we incubated Ramos cells with 50 µM of the corresponding compound for 16 h and subjected them to the Annexin V/7-AAD assay. Apoptosis was determined by detection of the phospholipid phosphatidylserine (PS) and its externalization on the cell surface. Compounds **15** and **16** increased the percentage of Annexin V positive cells from 4% to 57% and 39% respectively (Figure 5A). We further confirmed our observations by measuring DEVDase (caspase 3/7) activity. In Ramos cells incubated with 50 µM of inhibitors caspase 3/7-like activity peaked at 16 h and subsequently decreased (Figure 5B). In order to confirm the notion that compounds **15** and **16** induce caspase-dependent cell death, Ramos cells were pretreated in the presence or absence of zVad-fmk (50 µM), a pan-caspase inhibitor. Western blot analysis revealed that the procaspase-3 cleavage in Ramos cells induced with compounds **15** or **16** was completely blocked when cells were pretreated with zVad-fmk (Figure 5C). Additionally, zVad-fmk also suppressed compound-induced PS externalization. An increase in living cells from 62% to 82% as well as a decrease in Annexin V positive cells from 30% to 11% in the presence of the pan-caspase inhibitor were observed (representative data is shown for compound **16** in Figure 5D).

Taken together, these results demonstrate that compounds **15** and **16** are selectively cytotoxic and induce apoptosis in Ramos cells in a caspase-dependent manner.

### Disruption of Pro-survival Pathway in Burkitt’s Lymphoma through NFκB Inhibition

**Figure 6:**
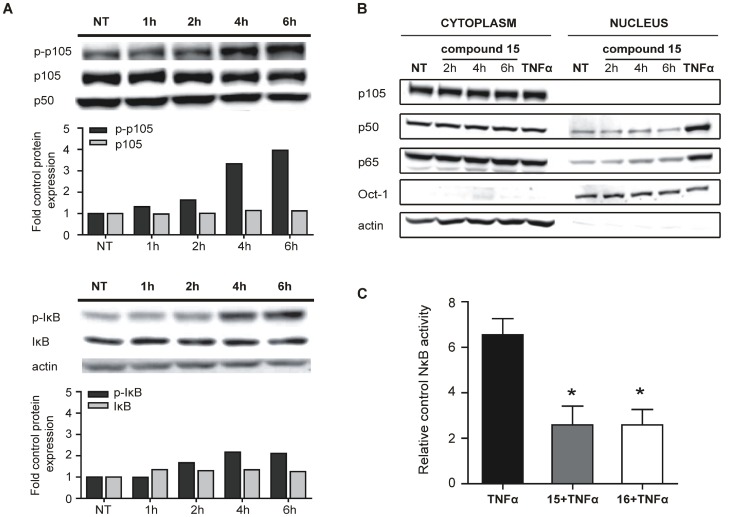
Accumulation of phospho-IκB and phospho-p105 with no subsequent increase in NFκB transcriptional activity. (A) Western blot analysis of Ramos cell lysates after treatment with compound **15** for the indicated time periods. Phospho-p105, p105, phospho-IκB and IκB were determined; with p50 or actin being used as loading controls. The relative quantifications of protein expression compared to control are graphically presented below each western blot. (B) Western blot analysis of cytoplasm and nuclear protein fractions after treating cells for indicated lengths of time with inhibitor **15** or TNFα (100 ng/mL, 6 h, the latter used as a positive control. (C) The determination of NFκB transcriptional activity. SEAP activity was measured in Ramos-Blue™ cells after pre-treating them for 1 h with compounds **15** or **16** (50 µM) and subsequently adding TNFα (100 ng/mL). After 8 h, the supernatant was collected and SEAP activity was determined. Results present 3 independent experiments. * P<0.05; NT, non-treated cells.

Since Ramos cells are known to constitutively express NFκB, an important cell survival signal, we chose to address the possible involvement of NFκB signalling modulation in the induction of cell death by serine protease inhibitors. Using Western blot analysis, we determined the levels of the NFκB precursor molecule p105 and the NFκB inhibitory molecule IκBα in Ramos cells treated with compound **15**. Although the treatment of cells caused the accumulation of the phosphorylated forms of p105 and IκBα, the total level of both molecules remained the same (Figure 6A). This indicates that the degradation of total p105 and IκBα with subsequent translocation of the p65/p50 unit did not occur. We confirmed this notion by performing subcellular fractionation and Western blot analysis on cytosolic and nuclear fractions of Ramos cells treated with compound **15** (50 µM) or TNF-α (100 ng/mL), The latter is used as positive control since it is a potent NFκB activator. We observed no translocation of p65 and p50 from the cytoplasm into the nucleus when cells were treated with compound **15** (Figure 6B). The observed inhibition of constitutive degradation of p105 and IκBα is expected to result in the stabilization of the p65/p50 heterodimer in the cytoplasm, leading subsequently to a decrease in the transcriptional activity of target genes. In order to obtain support for this assumption, we investigated NFκB transcriptional activity using Ramos-Blue™ cells, which stably express an NF-κB/AP-1-inducible secreted embryonic alkaline phosphate (SEAP) reporter construct. These cells were pretreated with the compound of interest (50 µM) for 1 h and subsequently stimulated with known NFκB activating stimuli, recombinant TNF-α (100 ng/mL). Determination of the SEAP activity in the supernatant after 8 h demonstrated that the presence of inhibitors **15** or **16** reduced TNF-α-induced NFκB activity by over 60% (Figure 6C). The data presented above indicate that the increased cell death caused by **15** or **16** could be due to the inhibition of NFκB activity followed by the disruption of pro-survival pathways in Burkitt’s lymphoma cells.

### Modulation of NFκB Signalling through Inhibition of Proteasome Activity

**Figure 7:**
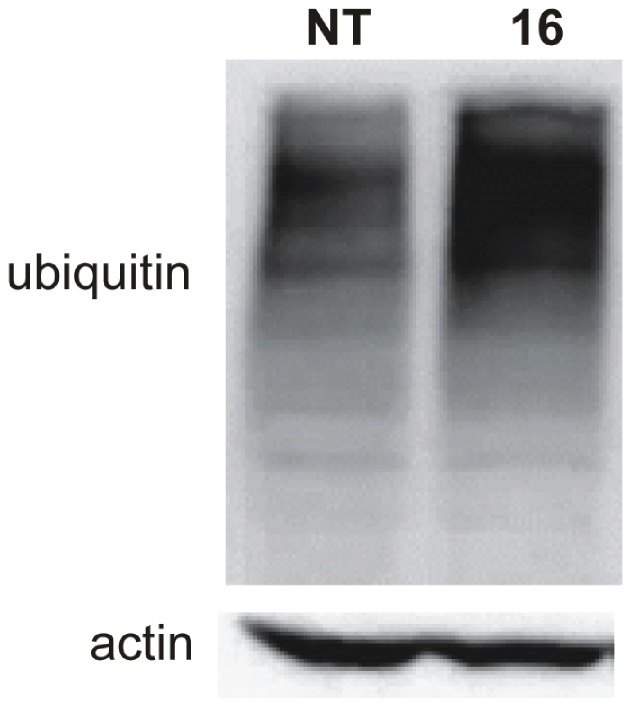
Inhibition of the proteasome. Western blot analysis of ubiquitinated proteins accumulation in Ramos cell protein extracts after 6 h of treatment with compound **16**. Actin was used as a loading control.

The accumulation of phospho-p105 and phospho-IκBα without subsequent NFκB activation led us to hypothesise that the tested serine protease inhibitors **15** and **16** could mediate their effects through the inhibition of proteasomal activities. With the objective of delineating this notion, Ramos cells were treated for 6 h with compound **16**, followed by a Western blot analysis of ubiquitinated proteins of high molecular mass. It is known that proteasomal inhibitors block the binding of ubiquitinated proteins to proteasome, resulting in their accumulation [28]. We determined that the treatment of Ramos cells with compound **16** resulted in an increased amount of polyubiquitinated proteins (Fig. 7), indicating that the inhibition of the proteasome had occurred.

In order to confirm that the accumulation of polyubiquitinated proteins was due to the inhibitory action of compounds on this multi-subunit complex, we performed a cell-free proteasome activity assay. We determined the K_m_ values for the substrates on the purified human 20S proteasome. For the chymotrypsin-, trypsin- and caspase-like activities the K_m_ values were 100 µM, 44 µM and 37 µM, respectively. Afterwards the mechanism of action and the inhibition constant for compounds **15** and **16** towards all three catalytic subunits of the proteasome were determined and are presented in Table 2. Using SigmaPlot 11 software we determined that both compounds inhibited the chymotrypsin- and caspase-like activities by an uncompetitive mechanism, while the trypsin-like activity was inhibited by a non-competitive mode of inhibition. As a positive control bortezomib was used. Its K_i_ were determined to be 1.5 nM for the chymotrypsin-like activity and >1000 nM for the trypsin- and caspase-like activities.

**Table 2:** K_m_ values of substrates and K_i_ values of compounds 15 and 16 for chymotrypsin-, trypsin- and caspase-like activities of human 20S proteasome.

20S proteasome activitiy	Substrate	K_m_ (µM)	Inhibitor	
			15	16
**Chymotrypsin-like**	Suc-Leu-Leu-Val-Tyr-AMC	100±12	K_i_ *'* = 84±15 µM	K_i_ *'* = 37±13 µM
**Trypsin-like**	Bz-Val-Gly-Arg-AMC	44±5	K_i_ = 13±5 µM	K_i_ = 17±7 µM
**Caspase-like**	Z-Leu-Leu-Glu-AMC	37±9	K_i_ *'* = 165±26 µM	K_i_ *'* = 70±3 µM

A rapid dilution assay for testing the reversibility of compounds, revealed an over 87% recovery of all three proteasomal activities, indicating that both compounds act as reversible inhibitors. Taken together, our results show that serine protease inhibitors **15** and **16** reversibly inhibit the proteasome, with the most prominent effect being observed on its trypsin-like activity.

### Docking Simulation

**Figure 8:**
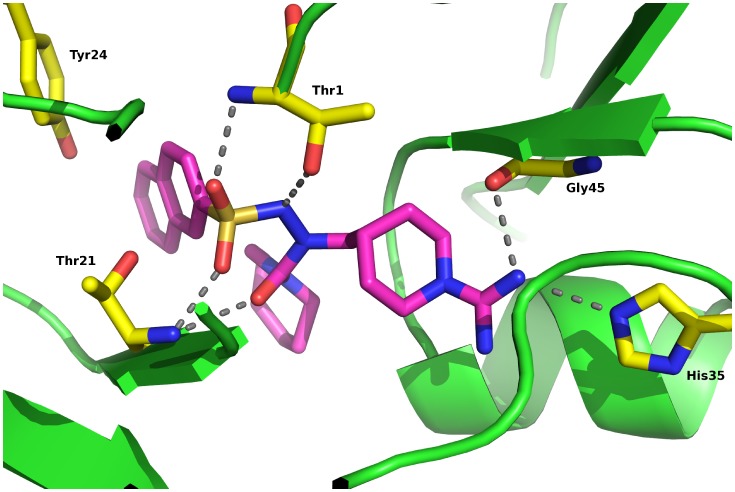
Molecular modelling. The binding mode of compound **16** in the trypsin-like (β2) subunit of the 20S proteasome. The compound is presented as a stick structure (carbon atoms are colored in pink, oxygen in red, nitrogen in blue and sulfur in yellow).

The plausible binding mode of compound **16** in the active site of β2-subunit was assessed by molecular docking, using crystal structure of yeast 20S proteasome in complex with bortezomib (PDB entry: 2F16) and LeadIT 1.3.0 software [29], [30]. In Fig. 8, the predicted binding conformation of compound **16** is shown. Compound **16** occupies similar region as the co-crystalized bortezomib, but some important additional interactions are predicted. Sulfonohydrazide moiety of **16** forms H-bonds with the catalytic residues Thr1 and Thr21, whereas its naphthalene ring forms π-stacking interactions with the Tyr24. Compound **16** fits well to S1-specificity pocket of β2-subunit, forming hydrophobic interactions with its piperidine ring. Additional polar interactions of the amidine group with His35 and Gly45 at the bottom of the S1-specificity pocket are also predicted.

## Discussion

Achieving selective cytotoxicity towards malignantly transformed cells is the main goal in the design and development of chemotherapeutic agents. In this study, we present amidinopiperidine-based serine protease inhibitors as selective inducers of apoptosis in Burkitt’s lymphoma cells. Their pharmacological effect is mediated through inhibition of the proteasome activity and the subsequent modulation of the NFκB pro-survival signalling.

Increased serine protease activity has been demonstrated in some cancers, making them potential new targets. For instance, the higher expression of the urokinase-type plasminogen activator has been determined in breast cancer and correlates strongly with greater cancer invasion and poor prognosis [31], [32]. It is similar in the case of tissue kallikreins (hK4–7), whose overexpression is reported to contribute to ovarian cancer progression [33]. In addition, the higher expression of certain endogenous serine protease inhibitors, such as maspin, is associated with the suppression of tumour growth and metastasis [34].

In our previous work, we examined the role of novel serine protease inhibitors as inducers of apoptosis on the murine and human B lymphoma cells WEHI 231 and Ramos, respectively, and identified a subgroup of compounds with an azaphenylalanine scaffold that exerted severe cytotoxicity [35]. A large planar hydrophobic naphthalene moiety proved to be crucial for the exertion of apoptosis; the selected compounds therefore reflect this property by possessing a *N'*-acyl-2-naphthohydrazide or *N'*-acyl-naphthalene-2-sulfonohydrazide scaffold. Cytotoxicity pre-screening of selected compounds was performed on the human B lymphoma, Ramos cells, and revealed a group of four compounds (**11**, **14**, **15**, **16**) that have a severe impact on the cell viability. When elucidating the correlation between the structure of the inhibitors and their biological response, we observed that the cytotoxic effect was particularly susceptible to the modifications of R^1^ residues and may be correlated to their basicity and structural features (Figure 2). Generally, the greater basicity of benzamidines, amidinopiperidines and piperidines resulted in an increased cytotoxic effect, an observation also made of certain slightly basic amidoximes (**1–3)** that are a well-known prodrug form of amidines. In most cases the introduction of a heteroatom in the six- or seven-membered rings at the R^2^ position (morpholine, piperazine) severely diminished the activity. The elongation of the structure caused by the introduction of the leucine moiety as an additional spacer between benzamidine and naphthalene also led to an inactive compound (**10**). Piperidine derivatives **11–16** generally exhibited increased cytotoxic activity compared to benzyl derivatives (**1–10)**. The former are nonplanar and the substituent on the piperidine nitrogen has a different spatial orientation and position. Transformation of the basic centre form piperidine (**10**) to amidinopiperidine (**15, 16**) does not influence the observed cytotoxic effect. However, compound **14,** with the nonbasic, acetamide moiety, showed comparable activity.

Selective cytotoxicity screening revealed that compounds **15** and **16** displayed a pronounced cell-type specific cytotoxic effect on the Burkitt’s lymphoma cells. The structural reasons for the greater cell type specificity could be explained by the presence of the amidinopiperidine fragment and its greater basicity relative to other inhibitors.

So as to delineate the molecular pathways involved in the mediation of the cytotoxic effects of inhibitors **15** and **16**, we looked into the NF-κB signalling pathway. The NF-κB family of transcription factors plays a key role in several cell processes, including the control of proliferation and apoptosis. Constitutive NF-κB activation is a common feature of most haematological malignancies and is believed to be crucial for the survival of these malignant cells. This is also the case in Ramos cells, where the strong pro-life signal mediated through NF-κB has been proposed as one of the reasons for their resistance to several chemotherapeutic agents [36], [37], [38]. Indeed, following the treatment with the serine protease inhibitor **15,** the NF-κB signalling pathway in Ramos cells was modulated.

These data indicated that inhibitors **15** and **16** may alter the proteasome activity. When the proteasomal activity is impaired, the ubiquitinated proteins (e.g. p105 and IκB) are not processed and, consequently, accumulate [28], [39]. This was also the case in Ramos cells incubated with compounds **15** or **16**. The proteasomal inhibition was additionally confirmed by an *in vitro* experiment. It showed a significant decrease in all three proteolytical activities of the purified human 20S proteasome; this was most notable in the case of the trypsin-like activity, with the K_i_ values of 13 µM and 17 µM for compounds **15** and **16,** respectively. Based on the docking simulation, we can postulate which structural features of amidinopiperidines are important for the proteasome inhibition. It seems that besides the H-bonding and the π-stacking interactions formed by the sulfonohydrazide moiety and the naphthalene ring, amidinopiperidine residue plays a crucial role to achieve notable inhibitory activity (piperidine by forming hydrophobic interactions and amidino group through polar interactions). As non-competitive mechanism of inhibition was determined for compound **16**, we can hypothesize that it binds to a different region of β2-subunit active site than artificial substrate. Such non-competitive inhibition by active-site binders is frequently observed with serine protease inhibitors [40]. Taken together, these data indicate that the inhibition of the proteasomal activity by serine protease inhibitors **15** and **16** is mediated through their direct binding to it. When looking at the time course, we observe that the proteasome inhibition occurs prior to the activation of the caspase cascade, indicating that the impairment of the NFκB-pathway is the main trigger for the induction of apoptosis.

The elucidation of molecular pathways identified proteasome as a target through which amidinopiperidine compounds induced cell death and as a potential reason behind the achieved selective cytotoxicity towards Burkitt’s lymphoma cells. These cells have a high proliferation rate, mutations in the NFκB leading to its constitutive activation, and have altered proteasomal activity due to variations in the subunit compositions. Thus they are more susceptible to the induction of apoptosis with proteasome inhibitors [41], [42], [43], [44]. Nevertheless, we do not exclude other targets of the amidinopiperidine compounds besides the proteasome. For instance, recent evidence suggests that other known proteasome inhibitors, such as bortezomib, also inhibit several other serine proteases (*e.g.* cathepsin G, HtrA2/Omi) [45].

In summary, we report of novel synthetic serine protease inhibitors as selective inducers of apoptosis in Burkitt’s lymphoma cells, where their effects are mediated through the inhibition of the proteasomal activity and subsequently modulate the NFκB signalling. The selective cytotoxicity of amidinopiperidine-based serine protease inhibitors **15** and **16** represents a good basis for the synthesis of new derivatives. Furthermore, they provide important tools for the investigation of the achieved selective cytotoxicity and the identification of a potential new target for anti-cancer therapy.

## Materials and Methods

### α-Chymotrypsin Inhibition Assay

The determination of K_m_ for α-chymotrypsin/Suc-Ala-Ala-Pro-Phe-AMC and inhibition constants for putative α-chymotrypsin inhibitors was performed as previously described [35], [46]. In short, a 1 nM solution of α-chymotrypsin was mixed with various concentrations of the substrate Suc-Ala-Ala-Pro-Phe-AMC. All the solutions were prepared in 144 mM Tris–HCl, pH 7.8. Substrate hydrolysis was monitored at 30°C on an automated Tecan Safire^2^ microplate reader (Tecan, Mannedorf/Zürich, Switzerland). The initial rates of the reactions (Δ*F*/Δ*t*) were measured immediately after the addition of the substrate (within 200 s) and plotted versus substrate concentration with nonlinear regression to fit a Michaelis-Menten plot. The curve fit and kinetic parameters (*K_m_* and *v*_max_) were calculated using GraphPad PRISM version 5.0 software (GraphPad Software, Inc., CA, USA). The recorded *K_m_* was the mean of six independent experiments (66±9 µM) and was used to calculate *K_i_* values for the tested compounds. The initial velocities of the hydrolysis reactions were measured under the same conditions as described for the determination of *K_m_*. 50 µl of α-chymotrypsin solution was added to 50 µl of each inhibitor solution (prepared in 144 mM Tris–HCl, pH 7.8; final concentration 1 nM) and incubated at 30°C for 15 min. The reaction was initiated with the addition of 100 µl of substrate. Each inhibitor was assayed at two concentrations (ranging from 3 to 100 µM, depending on solubility and accessibility) and at two substrate concentrations (50 µM and 100 µM); all the reactions were performed in triplicate. The *K_i_* values were determined as mean values of all measurements.

### Cell Culture

Cell lines Ramos, Daudi, U937 and Jurkat were purchased from ATCC (LGC Standards, UK), Ramos-Blue™ cells were from Invivogen (San Diego/CA, USA). Buffy coats from healthy volunteers were obtained by the Blood transfusion centre of Slovenia, according to institutional guidelines. Ramos, Daudi, U937 and Jurkat cell lines, as well as peripheral blood mononuclear cells (PBMC), were cultured in RPMI 1640 medium (Sigma-Aldrich, St.Luis/MO, USA) supplemented with 10% fetal bovine serum (Gibco, Grand Island/NY, USA), 2 mM L-glutamine, 100 U/ml penicillin, 100 µg/ml streptomycin and 50 µM 2-mercaptoethanol (all from Sigma-Aldrich) in a humidified chamber at 37°C and 5% CO_2_. Ramos-Blue™ cells were cultured in accordance with the manufacturer’s instructions.

### Metabolic Activity Assay

Cells (1×10^5^ cells/mL for Ramos, Daudi, U937 and Jurkat, and 1×10^6^ cells/mL for PBMC) were treated with the appropriate amounts of compounds of interest or corresponding vehicle (control cells), then seeded in triplicate in 96 well plates. The metabolic activity was assessed using the CellTiter 96® Aqueous One Solution Cell Proliferation Assay (Promega, Madison/WI, USA), in accordance with the manufacturer’s instructions.

### Cell Cycle Analysis

Ramos cells (5×10^5^ cells) were treated with the compound of interest for 24 h. The cells were then washed with PBS and subjected to fixation with 80% ethanol at –20°C for 15 min. Fixated cells were pelleted by centrifugation and rehydrated in 5 mL of PBS for 15 min at room temperature. Collected cells were resuspended in 500 µL staining buffer (3 µM propidium iodide, 100 mM Tris, pH 7.4, 150 mM NaCl, 1 mM CaCl_2_, 0.5 mM MgCl_2_, 0.1% Nonident P-40). After 15 min of incubation, samples were analysed on a FACScalibur and evaluated using FlowJo software (BD bioscience, Franklin Lakes/NJ, USA).

### Annexin V Assay

We used a PE Annexin V Apoptosis Detection Kit I (BD Biosciences) in accordance with the manufacturer’s instructions. Put briefly, Ramos cells were, following their treatment with the compound of interest, washed with cold PBS and resuspended in 1× binding buffer at a 1×10^5^ cells/mL concentration. 100 µL of cell suspension was transferred to a 5 mL tube and 5 µL of both PE Annexin V and 7-AAD was added. The suspension was gently vortexed and incubated for 15 min at room temperature in the dark. Following this, 400 µL of 1× binding buffer was added and samples were analysed using a FACScalibur flow cytometer.

### Determination of Caspase 3/7 Activity

Caspase 3/7 activity was measured in total cell lysates utilizing a fluorescent DEVD substrate and the incubation of cells with 10 µM TPCK as positive control as described previously [19]. The process can be summarised as follows. Having been incubated with the chemicals for the indicated amounts of time, cells (2×10^6^) were washed twice in PBS and resuspended in 200 µl of ice-cold caspase lysis buffer (0.1% Triton X-100, 100 mM phosphate buffer, pH 6.0, 1.3 mM EDTA, 100 mM NaCl), sonicated and left on ice (30 min). After centrifugation (14,000× g, 15 min, 4°C), the total protein concentration in the supernatants was measured using the BioRad Protein Assay Kit (Bio-Rad, Hercules/CA, USA), in accordance with the manufacturer’s instructions. Cell lysates (20 µg of protein) were incubated for 30 min at 37°C in caspase reaction buffer (20 mM PIPES, pH 7.2, 10% sucrose, 0.1% CHAPS, 1 mM EDTA, 100 mM NaCl), after which 100 µM Ac-DEVD-AFC peptide substrate (Bachem, Bubendorf, Switzerland) was added. Immediately following the addition of the substrate, fluorescence intensity was monitored continuously for 30 min using a fluorescence microplate reader (Tecan Safire^2^) at excitation and emission wavelengths of 405 and 535 nm respectively. Data were expressed as the increase in fluorescence as a function of time (ΔF/Δt).

### Western Blot Analysis

Ramos cells were seeded in 6-well culture plates at a concentration of 1×10^6^ cells/mL and treated with the compound of interest or corresponding vehicle for 1 h, 2 h or 6 h. After the indicated time points, cells were harvested, washed in ice-cold PBS and lysed in modified RIPA buffer (50 mM Tris-HCl, pH 7.4, 150 mM NaCl, 1% NP−40, 0.25% Na-deoxycholate, 1 mM EDTA, 1 µg/mL aprotinin, 1 mM PMSF and 1× Halt Phosphatase inhibitor cocktail (Thermo Scientific, Pierce Biotechnology, IL, USA)). The lysates were sonicated, rocked on ice for 30 min and centrifuged at 13,000 rpm at 4°C for 15 min. Protein samples were electrophoresed in SDS-polyacrylamide gels and then transferred to nitrocellulose membranes. The primary antibodies used were against ubiquitin, phospho-IκBα, IκBα, p65 and caspase-3 (Cell Signaling, Danvers/MA, USA), and p105/p50 (Santa Cruz Biotechnology, Inc., Santa Cruz/CA, USA), and ß-actin (Sigma-Aldrich). Following incubation with the primary antibody, membranes were washed six times and incubated for 1 h at room temperature with the corresponding dilution of the appropriate secondary antibody conjugated with horseradish peroxidase (Upstate, Temecula, CA, USA). The immunoreactivity of respective proteins of interest was determined by chemiluminescence using the SuperSignal West Femto substrate (Pierce, IL, USA), in accordance with the manufacturer's instructions. To ensure the equal loading of proteins, the membranes were stripped and reprobed with appropriate antibodies under the same conditions as those described above.

### Subcellular Fractionation

Ramos cells were harvested by low centrifugation at 4°C and washed twice with ice-cold PBS. They were resuspended in 250 µL of lysis buffer A (10 mM HEPES, pH 7.9, 1.5 mM MgCl_2_, 10 mM KCl, 0.1% Nonident P-40, 0.5 mM DTT, 1 mM PMSF), vortexed for 10 s and incubated on ice for 10 min. The supernatant with cytoplasmic fraction was collected after centrifugation at 5,200 rpm for 10 min at 4°C. The pellet was resuspended in 100 µL of lysis buffer B (20 mM HEPES, pH 7.9, 1.5 mM MgCl_2_, 420 mM NaCl, 0.2 mM EDTA, 25% glycerol, 1 mM PMSF) and incubated on ice for 1 h, with occasional vigorous vortexing. The nuclear extract was recovered by centrifugation at 16,000 rpm for 10 min at 4°C.

### Quanti-blue Assay

Ramos-Blue™ cells (Invivogen), which stably express an NF-κB/AP-1-inducible secreted embryonic alkaline phosphate (SEAP) reporter construct, were assayed for NFκB transcriptional activity changes upon pretreating them with the compound of interest (50 µM) for 1 h and subsequently stimulating them with recombinant TNF-α (100 ng/mL). SEAP activity was determined in the supernatant in accordance with the manufacturer’s instructions. Briefly, to 200 µL of QUANTI-Blue regent 20 µL of cell supernatant was added and incubated at 37°C for 3 h. Abosorbance was measured on microplate reader Tecan Safire^2^ at 640 nm.

### Proteasome Activity Measurements

K_m_ was determined by mixing 1 µg/mL of the purified human 20S proteasome (Boston Biochem, Inc., Cambridge/MA, USA) with various concentrations of the corresponding substrate ranging from 5 µM to 600 µM. Suc-Leu-Leu-Val-Tyr-AMC, Bz-Val-Gly-Arg-AMC and Z-Leu-Leu-Glu-AMC (Bachem, Bubendorf, Switzerland) were used for the chymotrypsin-like, trypsin-like and caspase-like activites, respectively. Assay buffer consisted of TE buffer (20 mM Tris (pH = 8.0), 0.5 mM EDTA, 0.03% SDS). Substrate hydrolysis was continuously monitored at 37°C with an automated microplate reader (Tecan Safire^2^). Measured fluorescence values (RFU) were plotted versus time with nonlinear regression to fit a Michaelis-Menten plot.

For the K_i_ determination, 25 µL of substrate at three concentrations and 25 µL of inhibitor of interest also at three different concentrations were added to the wells of a white microplate. The reaction was initiated with 50 µL of the enzyme in the assay buffer. Flourescence of the AMC release was continuously monitored at 420 nm with 320 nm excitation.

K_m_ and K_i_ were calculated using SigmaPlot 11 (Enzyme Kinetics Module 1.3) and are presented as the mean of three independent experiments.

### Rapid Dilution Assay

20S proteasome at 100-fold final concentration (100 µg/mL) and inhibitor at 10-fold IC_50_ were incubated for 30 min at room temperature at 2 µL. The enzyme-inhibitor complex was diluted 100-fold with the substrate of interest to a final volume of 200 µL, initiating the reaction. In the case of reversibility of the inhibitor the residual enzyme activity was expected to be around 90%.

### Molecular Modelling

The computational study was carried out on a workstation with 4 dual-core AMD Opteron processors, 16 GB of RAM, a GeForce 7800 graphics card, and 1.2 TB of hard disk space, running the 64-bit Fedora 7 version. For the docking simulation, LeadIT 1.3.0 (BioSolveIT GmbH) was used on the crystal structure of the yeast 20S proteasome in complex with bortezomib (PDB entry: 2F16) [29], [30]. Based on the kinetic studies the active site was defined as the area within 20Å of the N-terminal Thr1 residue of β2-subunit. Protonations and OH group orientations of the active-site amino acid residues were manually assigned with LeadIT GUI. For base placement Triangle Matching was used and the program generated maximally 200 solutions per iteration and 200 per fragmentation. We validated the system by the re-docking of co-crystallized bortezomib. For figure preparation the PyMOL Molecular Graphics System Version 1.3 was used (Schrödinger, LLC).

### Statistical Analyses

Results are presented as means ± SD of three independent experiments. Statistical comparisons between groups were evaluated using Student’s *t* test, where *p*<0.05 was considered statistically significant.
